# Precision Plasma Electrolytic Polishing of GH3536 Superalloy for Effective Surface Performance Improvement

**DOI:** 10.3390/ma19061127

**Published:** 2026-03-13

**Authors:** Chengtao Peng, Siqi Wu, Xinming Wang, Chen Zhang, Jing Sun, Jinlong Song

**Affiliations:** 1 State Key Laboratory of High-Performance Precision Manufacturing, Dalian University of Technology, Dalian 116024, China; 18101536086@163.com (C.P.);; 2 Key Laboratory for Micro/Nano Technology and System of Liaoning Province, Dalian University of Technology, Dalian 116024, China; 3 School of Mechanical Engineering, Dalian University of Technology, Dalian 116024, China

**Keywords:** plasma electrolytic polishing, GH3536 superalloy, low roughness

## Abstract

GH3536 superalloy is widely used in the high-temperature components of aerospace applications for its excellent high-temperature strength and corrosion resistance. However, under such a harsh environment, surface defects can make the superalloy prone to corrosion and fatigue fractures. Therefore, it is important to eliminate surface defects through polishing. However, the existing polishing methods usually suffer from some issues such as surface integrity damage, low efficiency, and poor environmental sustainability. More importantly, these methods fail to account for the requirement of surface roughness below 0.05 μm in some high-precision aerospace components. Herein, the plasma electrolytic polishing (PEP) of GH3536 superalloy is systematically investigated and optimized through single-factor experiments and response surface methodology (RSM). A minimum surface roughness *Ra* of 0.044 μm with a mirror-like surface was achieved at a voltage of 303.8 V, electrolyte temperature of 66.2 °C, polishing time of 5 min, and submersion depth of 7.5 cm. At the same optimized condition, the material removal rate was 59.12 mg·min^−1^. After polishing, the surface composition of GH3536 superalloy varied negligibly, while its corrosion resistance improved markedly, with a 53.72% increase in polarization resistance and a 43.46% decrease in corrosion current density. Meanwhile, the microhardness slightly decreased due to the removal of the work-hardened layer and the compressive residual stress exhibited a more uniform distribution across the surface, contributing to improved near-surface mechanical stability. This study establishes an optimized PEP parameter for improving the surface quality of GH3536 superalloy, offering a practical method for the precision finishing of aerospace-grade superalloy components.

## 1. Introduction

GH3536 superalloy, with its outstanding high-temperature strength, creep resistance, corrosion resistance, and thermal stability, has become a key material for aircraft engine hot-end components, nuclear reactor systems, and high-temperature chemical equipment [1,2,3]. These components typically operate under harsh conditions of high temperature, high pressure, and cyclic high loads [4]. Under such demanding environments, any machining defect on their surface can lead to stress concentration and crack initiation, eventually causing fatigue fracture [5]. Therefore, to ensure the reliability and durability of these components, it is important to eliminate these surface defects through polishing. To date, researchers have proposed several polishing methods, including mechanical polishing [6], abrasive flow machining [7,8], magnetorheological polishing [9,10], chemical polishing [11,12], electropolishing [13,14,15], and plasma electrolytic polishing [16,17]. However, mechanical polishing is prone to introducing surface and subsurface damage because of the alloy’s inherent high hardness, high toughness, and complex multiphase microstructure [18,19,20]. Additionally, mechanical polishing typically requires a large amount of manual labor and time to achieve higher surface quality, resulting in low efficiency. Moreover, it is difficult to mechanically polish components with complex geometries due to a lack of tool accessibility to all surfaces [21,22]. Abrasive flow machining can address internal channel structures but is limited by the high wear resistance of GH3536 superalloy, resulting in low polishing efficiency and an uneven surface finish [7]. Magnetorheological polishing offers high precision, but the cost is high. Additionally, the polishing efficiency for ultra-high-strength GH3536 superalloy is low because of insufficient shear stress. Chemical polishing and electropolishing can polish complex-shaped components without introducing stress or strain, but the use of high-concentration and strongly corrosive solutions poses safety hazards and complicates waste disposal [17,23].

Among these polishing methods, plasma electrolytic polishing (PEP) has been widely studied as an efficient and environmental friendly polishing method that does not introduce stress or strain [24]. In the PEP process, a non-toxic low-concentration salt solution is used as the electrolyte, and the sample serves as the anode. By applying an appropriate direct current (DC) voltage, the electrolyte near the anode evaporates due to Joule heating, while an oxygen evolution reaction occurs at the anode, forming a vapor–gas envelope (VGE) [25]. This VGE has high resistivity and generates a strong electric field within it. The VGE is then ionized and undergoes dielectric breakdown, forming local discharge channels that produce plasma and induce complex physicochemical reactions on the sample surface [26]. Microscopic protrusions on the sample surface exhibit higher local electric field intensity and are more likely to form discharge channels and be preferentially removed, thereby achieving selective removal of surface peaks and overall surface leveling [27]. This material removal mechanism enables PEP to effectively overcome the limitations of the aforementioned methods. Specifically, PEP avoids mechanical damage, handles complex geometries easily, operates conveniently, and requires no corrosive chemicals, all while maintaining a high polishing efficiency. These advantages make it particularly suitable for hard materials such as GH3536 superalloy.

At present, PEP has been shown to yield excellent polishing results on various metals and alloys [16]. For example, Zhou et al. combined experiments and a simulation to analyze the evolution of the VGE and explained how the VGE characteristics affect the surface morphology and polishing efficiency from an energy distribution perspective. Their experiments showed that discontinuous oscillating VGEs facilitated material removal and surface finishing [22]. K. Sushil et al. investigated the microstructural features and related surface properties of Inconel 718 after PEP. The average surface roughness *Ra* was reduced from 0.238 μm to 0.067 μm, yielding a smoother optical reflective surface with a slightly decreased friction coefficient and wear rate, a slight drop in Vickers hardness, and improved wettability [28]. These studies provide valuable insights into the fundamental physicochemical processes of PEP. However, since their conclusions are derived from other alloys, the applicability of the reported process rules to GH3536 superalloy, with its distinctly different microstructure, remains to be verified.

Recently, researchers have begun to apply PEP to GH3536 superalloy. Wu et al. developed a spray electrolytic plasma polishing (SEPP) method for large complex surfaces, reducing the surface roughness of a selective laser-melted (SLM) GH3536 alloy plate from *Ra* = 13.93 μm to 0.107 μm. This process removed oxides and other impurities from the sample surface and increased the relative contents of Ni, Cr, and Fe [29]. Yan et al. applied an internal electrode PEP technique to additively manufactured nickel-based superalloy samples to improve their surface quality. In their work, the surface roughness of a GH3536 sample with internal channels decreased from 15.1 μm to 2.793 μm [30]. These studies have strongly demonstrated the feasibility of PEP for GH3536 superalloy, showing significant engineering application value. However, the reported final roughness of more than 0.1 μm remains insufficient for the stringent roughness requirement of less than 0.05 μm demanded by certain high-precision aerospace applications. Therefore, it is extremely necessary to develop an efficient and environmentally friendly polishing method for GH3536 superalloy that induces no surface integrity damage. Importantly, this method needs to meet the requirement of a surface roughness of less than 0.1 μm for certain high-precision aerospace components.

To address these limitations, this study presents a systematic optimization of PEP to achieve surface roughness values below 0.05 μm for GH3536 superalloy. While previous work polished original surfaces with 0.1–2.7 μm roughness, we focus on surfaces with a pre-treated roughness of approximately 0.5 μm. Initially, the independent effects of the voltage, polishing time, electrolyte temperature, and submersion depth on the surface roughness and material removal rate were analyzed through single-factor experiments systematically. Subsequently, we obtained a mirror-like surface with an optimal surface roughness of 0.044 μm at a voltage of 303.8 V, electrolyte temperature of 66.2 °C, polishing time of 5 min, and submersion depth of 7.5 cm, after further analyzing the influence of the PEP parameters and optimizing them through response surface morphology (RSM). Based on the optimized parameters, the macro- and micro-scale surface morphologies, phase composition, surface elemental composition and corrosion resistance of the samples before and after PEP were compared and analyzed to show the enhanced surface performance.

## 2. Materials and Methods

### 2.1. Materials

The samples used in this experiment were GH3536 superalloy plates with dimensions of 30 mm × 20 mm × 1 mm. To ensure a uniform initial surface roughness, the surfaces were ground with 180-grit metallographic abrasive paper until uniform roughness of about 0.5 μm was achieved. The samples were then ultrasonically cleaned in absolute ethanol, rinsed with deionized water, and dried. These prepared specimens were recorded as the initial samples.

### 2.2. PEP Process

**Figure 1:**
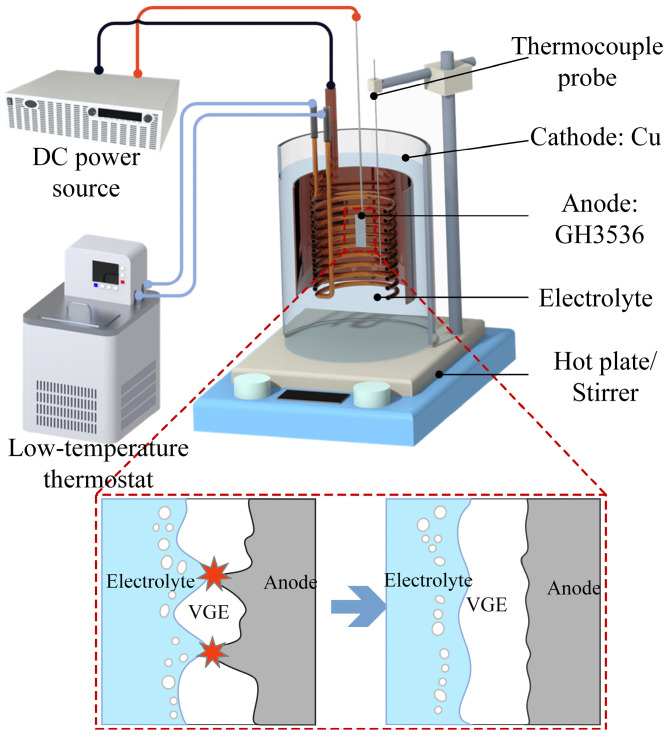
Schematics of the plasma electrolytic polishing devices and mechanism.

The experimental setup for the plasma electrolytic polishing (PEP) process is schematically illustrated in Figure 1. A 30 kW programmable DC power supply that could be connected to a computer for real-time acquisition of the current and voltage data was used. A brass coil connected to a cooling fluid circuit was placed in the electrolyte cell and circulated with coolant from an external low-temperature constant-temperature bath, maintaining the electrolyte temperature at the set value (±1 °C). A copper plate with a size of 1 mm × 50 mm × 380 mm was bended and used as the cathode. The area ratio of the cathode to anode was greater than 10:1 to ensure a stable VGE formation on the anode surface [17]. The sample served as the anode, and after applying the voltage, a motorized lifting platform was used to control the submersion depth. A magnetic stirrer at the bottom of the cell was used to stir the electrolyte. Based on the literature and preliminary experiments, the electrolyte solution was prepared using 4 wt% ammonium sulfate and 1 wt% sodium citrate. Ammonium sulfate was selected as a supporting electrolyte to provide sufficient ionic conductivity and ensure stable current distribution during the PEP process. Sodium citrate was introduced as a complexing agent to regulate anodic dissolution and reduce possible surface deposition by forming soluble complexes with metal ions. The processing parameters significantly affected the surface quality of the sample. To optimize the processing parameters, single-factor experiments were first conducted to investigate the influence of the voltage, electrolyte temperature, polishing time, and submersion depth (distance between the sample’s upper edge and the electrolyte’s liquid level) on surface roughness and material removal rate. Based on the results of the single-factor experiments, appropriate parameter ranges were selected. These ranges were then further optimized using response surface methodology (RSM). The optimized processing parameters were finally validated through confirmatory experiments.

### 2.3. Characterization

The surface roughness of the samples was tested according to a surface roughness measuring instrument (JD520, Beijing Ji Tai Tech Detection Device, Beijing, China). To minimize local measurement errors caused by surface inhomogeneity, ten measurements were carried out at different positions on each sample. The maximum and minimum values were excluded, and the average of the remaining eight values was taken as the final surface roughness of the sample. The 3D surface profile was analyzed using a white-light interferometer (WLI, NV5000 5022S, ZYGO, Middlefield, CT, USA). The surface morphology of the samples was observed using a field emission scanning electron microscope (SEM, SEM5000, National Instruments, Beijing, China) equipped with an energy-dispersive spectroscope (EDS, INCAs energy, Oxford Instruments, Abingdon, UK). The phase composition of the samples was analyzed by an X-ray diffractometer (XRD, D8 ADVANCE, Bruker (Beijing) Scientific Technology, Berlin, Germany). The samples were scanned from 10°to 90° with a step size of 0.02° at a scanning rate of 2°·min^−1^. The corrosion resistance tests were performed using an electrochemical workstation (PARSTAT 3000-DX, AMETEK, Inc., Berwyn, PA, USA). A 3.5 wt% NaCl solution served as the electrolyte. The open-circuit potential (OCP) was first measured and recorded for 1800 s to ensure the stability of the samples in PBS solution. After the OCP measurements, electrochemical impedance spectroscopy analysis was performed running a 10 mV peak-to-peak sinusoidal voltage signal in the 10^−2^ to 10^5^ Hz frequency range. Dynamic potential polarization tests were set with a potential scanning rate of 0.1666 mV·s^−1^ and a potential range of −0.25 V to 0.25 V. An analytical balance (precision: 0.1 mg) was used to determine the quality degradation of the sample during polishing. The Vickers hardness of the samples was tested by a Vickers hardness tester (MVA-301, DINGPA, Shenzhen, China). The residual stress of the samples was tested by an X-ray stress analyzer (XL-640, Handan Stress Technologies, Handan, China).

## 3. Results and Discussion

### 3.1. Processing Parameters of PEP

#### 3.1.1. Single-Factor Analysis

**Figure 2:**
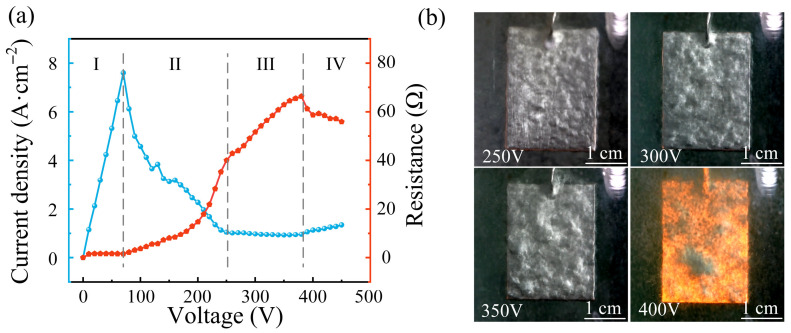
The influence of voltage on PEP progress: (**a**) current density and resistance in different voltage regions, and (**b**) polishing phenomenon in different voltage regions.

**Figure 3:**
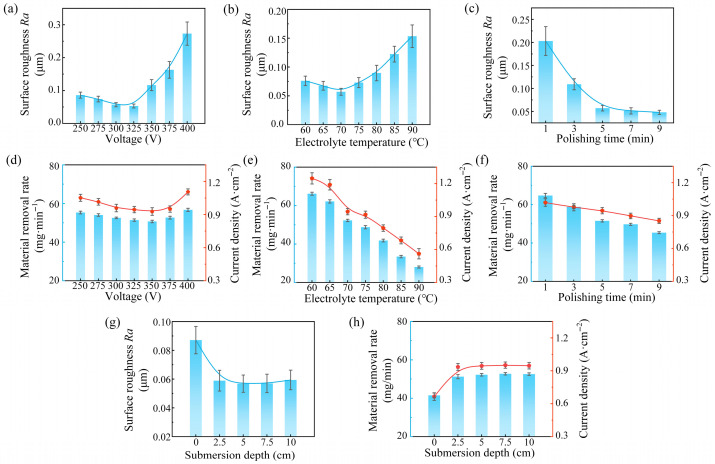
The influence of voltage, electrolyte temperature, polishing time, and submersion depth on surface roughness, material removal rate, and current density: (**a**) the variation in surface roughness with the voltage, (**b**) the variation in surface roughness with electrolyte temperature, (**c**) the variation in surface roughness with the polishing time, (**d**) the variation in material removal rate and current density with the voltage, (**e**) the variation in material removal rate and current density with the electrolyte temperature, (**f**) the variation in material removal rate and current density with the polishing time, (**g**) the variation in surface roughness with the submersion depth, (**h**) the variation in material removal rate and current density with the submersion depth.

Since all processing parameters, including the voltage, electrolyte temperature, polishing time, and submersion depth, affect the surface roughness and material removal rate during the PEP process, a series of single-factor experiments was conducted. Among these processing parameters, the voltage played a decisive role in the PEP process. Therefore, we investigated the relationship between the voltage and both current density and resistance by applying a linearly increasing voltage and fitting the results. As shown in Figure 2a, this curve can generally be divided into four stages. Stage I was the electrolytic stage, where Faraday’s law applies, meaning that the current density increased with the increasing voltage while the resistance remained constant. As the voltage increased into stage II, many gas bubbles formed on the sample surface. These bubbles hindered the direct contact between the sample and the electrolyte, causing a sharp increase in the resistance and a sharp decrease in the current density. When the gas generated by the electrolysis integrated with the vapor generated by ohmic heating to form a stable VGE with sufficient thickness, the system entered the PEP stage (stage III). As illustrated in Figure 1, a strong electric field was formed within the vapor layer enveloping the sample. Surface geometry influences the electric field distribution. Asperities with smaller curvature radii may induce local electric field amplification, which can increase the probability of dielectric breakdown at protrusions. As a result, micro-discharges are more likely to occur at surface peaks, where transient high-energy input and enhanced anodic dissolution promote preferential material removal. As polishing proceeds, the peak height decreases and the surface curvature becomes more uniform, leading to a more homogeneous electric field distribution and reduced discharge localization. This dynamic feedback mechanism contributes to progressive surface smoothing. As shown in Figure 2b, at the 250 V critical voltage for entering the PEP stage, the VGE was relatively thin, and the abrasive paper scratches on the sample surface were still seen. As the voltage increased further, the VGE thickness and resistance increased, while the current density remained nearly constant. When the voltage reached approximately 400 V, the system entered the arc discharge stage (stage IV) with intense arc light and high-frequency noise. The sample surface was surrounded and violently bombarded by the luminous plasma, resulting in poor polishing. Therefore, single-factor experiments were conducted at seven voltage levels from 250 V to 400 V in 50 V increments while keeping the electrolyte temperature, polishing time held, and submersion depth constant at 70 °C, 5 min, and 7.5 cm, respectively. As shown in Figure 3a, the surface roughness of the samples first decreased from 0.086 μm to 0.053 μm and then increased to 0.273 μm with an increasing voltage from 250 V to 400 V, reaching a minimum surface roughness of 0.053 μm at 325 V. Figure 3b shows that the current density and material removal rate also first decreased and then increased with the increasing voltage, but the variations were relatively small.

The electrolyte temperature was another critical parameter in the PEP process, primarily through its influence on the VGE. The formation of this VGE depended on the vigorous boiling of the electrolyte attached to the sample surface. At a given current density, a higher electrolyte temperature required less energy to reach the boiling point, facilitating bubble generation and coalescence into a VGE. Conversely, at a lower temperature, a higher current density was needed to generate and maintain the VGE. Beyond its direct effect on VGE generation, the electrolyte temperature also affected the electrolyte conductivity, viscosity, and surface tension. In addition, the electrolyte temperature affected the critical voltage for the transition from stage II to stage III of the PEP process [22]. Given these multiple effects of the PEP process, identifying an optimal electrolyte temperature is essential. Therefore, we first conducted pre-experiments whose results revealed that stable polishing could not be achieved below an electrolyte temperature of 60 °C, while temperatures exceeding 90 °C caused vigorous boiling and splashing, leading to poor polishing. Based on the above results, single-factor experiments were conducted at seven temperature levels from 60 °C to 90 °C in 5 °C increments, with the voltage and polishing time kept constant at 325 V and 5 min, respectively. As shown in Figure 3c, the surface roughness first decreased from 0.076 μm to 0.053 μm and then increased to 0.153 μm as the temperature increased from 60 °C to 90 °C, reaching a minimum surface roughness of 0.053 μm at 70 °C. Figure 3d shows that both the current density and material removal rate decreased monotonically with the increasing temperature. This is because higher electrolyte temperatures caused a thicker VGE on the anode surface, thereby increasing the resistance of the VGE, decreasing the current density, and decreasing the material removal rate [31].

We then investigated the influence of polishing time on the PEP process. As shown in Figure 3e, the surface roughness decreased rapidly from 0.203 μm to 0.053 μm as the polishing time increased from 1 min to 5 min at first, and then decreased extremely slowly from 0.053 μm to 0.048 μm as the time increased from 5 min to 9 min. Concurrently, Figure 3f shows that both the current density and material removal rate decreased monotonically with the time, which was attributed to the evolution of surface geometry. Initially, the sharp protrusions on the surface caused a tip effect, concentrating the electric field and enabling rapid dissolution. As the surface flattened, the electric field uniformized, weakening the material removal rate.

Next, the influence of submersion depth on the PEP process was also investigated. As shown in Figure 3g, the surface roughness was highest at 0.087 μm at a 0 cm submersion depth, while the surface roughness remained unchanged at about 0.054 μm as the submersion depth increased from 2.5 cm to 10 cm. Simultaneously, both the current density and material removal rate at a 0 cm submersion depth were lower than that at other submersion depths. Both the current density and material removal rate remained unchanged as the submersion depth increased from 2.5 cm to 10 cm (Figure 3h). This trend was due to inadequate electrolyte coverage and instability of the VGE. As a result, the local discharges at the top surface were frequently interrupted, leading to increased surface roughness, reduced current density, and reduced material removal rate. Consequently, the influence of the individual processing parameters on both the surface roughness and material removal rate was successfully determined through the single-factor experiments.

#### 3.1.2. Response Surface Methodology Optimization of PEP Parameters

Based on the single-factor experiments, the surface roughness decreased by 88.6% when the polishing time increased from 0 min to 5 min, but only decreased by 1.8% when further increasing the time from 5 min to 9 min. For the submersion depth, except for 0 cm, the surface roughness varied by less than 4.5% across the other depths, indicating a negligible influence. Therefore, only voltage and electrolyte temperature, which showed substantial effects on surface roughness and material removal rate, were included in the multivariate RSM optimization. A central composite design was employed for response surface analysis with voltage and temperature ranges of 275–350 V and 60–80 °C, respectively. Thirteen experiments, including five replicates at the center point, were performed to construct a second-order model, thereby reducing the number of full-factor experiments and capturing the effects of the nonlinear interactions between the voltage and temperature on the surface roughness and material removal rate while identifying the optimal surface roughness. The experimental results are shown in Table 1.

Based on these experimental results, quadratic regression models were developed to describe the relationship between the processing parameters and the two responses: surface roughness *Ra* and material removal rate.

For *Ra*, the fitted quadratic regression model using coded factors was

(1) $$Ra=0.0484+0.0199A+0.0099B+0.008AB+0.0277A^{2}+0.0121B^{2}$$

where *A* and *B* denote the coded values of voltage and electrolyte temperature, respectively.

The ANOVA results of the quadratic response surface model for the *Ra* model are summarized in Table 2.

The ANOVA results confirm that the model was highly significant, with *F* = 811.92 and *p* < 0.0001. All terms, including the *AB* term with *F* = 103.34 and *p* < 0.0001, were statistically significant. The lack of fit was not significant, with *p* = 0.1507, indicating that the quadratic model adequately described the experimental data.

The positive coefficient of the *AB* term revealed a synergistic interaction between the voltage and temperature, indicating that the interaction effect on surface roughness was slightly amplified as both parameters increased. This interaction can be further interpreted through partial derivatives, which quantify how each factor responds to changes in the other. The partial derivatives were

(2) $$\frac{\partial Ra}{\partial A}=0.0199+0.008B+0.0554A$$

(3) $$\frac{\partial Ra}{\partial B}=0.0099+0.008A+0.0242B$$

The partial derivatives show that the instantaneous effect of the voltage depended on the current temperature, and vice versa. At the design center, the effect of voltage was twice that of temperature. This disparity widened at the high–high corner, demonstrating how the positive *AB* term amplifies their sensitivities. At the optimal condition, the marginal effects were small ($\frac{\partial Ra}{\partial A}=0.0046$,$\frac{\partial Ra}{\partial B}=-0.0011$), which is consistent with a local extremum where both factors exerted minimal influence.

The model’s predictive capability is evident from the fit statistics: R^2^ = 0.9983, adjusted R^2^ = 0.9970, and predicted R^2^ = 0.9906, indicating that the model explains nearly all variations and can extrapolate reasonably without overfitting. An adequate precision value of 78.1517 confirms a strong signal-to-noise ratio, allowing the design space to be navigated reliably.

**Figure 4:**
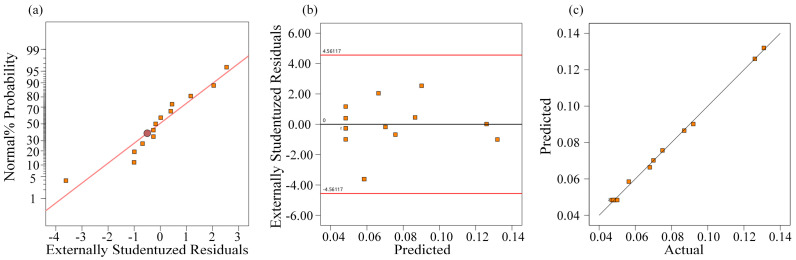
Residual diagnostics for the regression model of *Ra*: (**a**) normal probability plot of residuals, (**b**) residuals versus predicted, and (**c**) predicted versus actual.

The normal probability plot of residuals (Figure 4a) shows that the residuals followed an approximately linear distribution, indicating normality. The residuals versus predicted plot (Figure 4b) displays random dispersion without obvious patterns, suggesting homoscedasticity. The predicted versus actual (Figure 4c) plot demonstrates strong agreement between the experimental and predicted values. These results confirm the robustness and reliability of the regression model.

**Figure 5:**
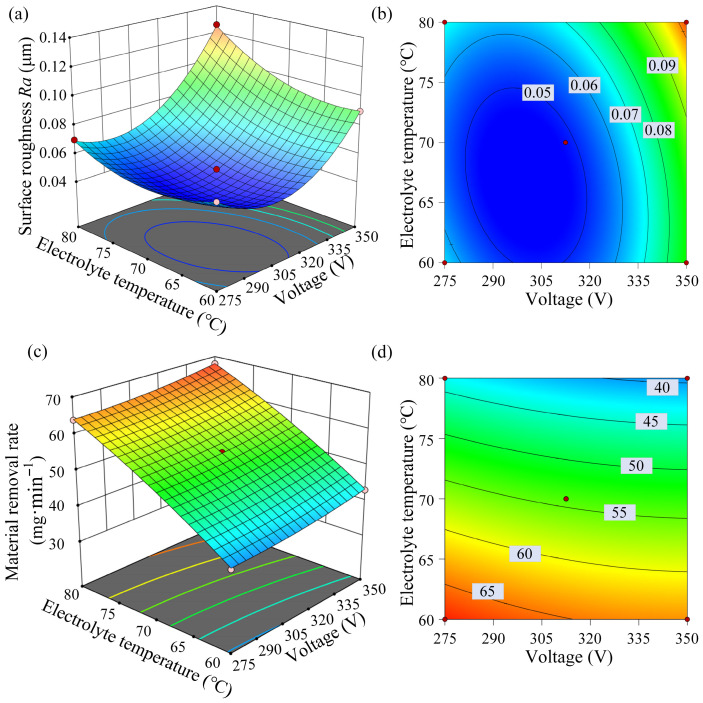
The 3D response surface and contour plots: (**a**) 3D response surface for surface roughness *Ra*; (**b**) contour plot for surface roughness *Ra*; (**c**) 3D response surface for the material removal rate; (**d**) contour plot for the material removal rate.

The interactive effects between the voltage and temperature on the surface roughness are illustrated in a 3D response surface plot and corresponding contour plot (Figure 5a,b). The plots clearly show that the surface roughness first decreased and then increased as the voltage and temperature increased, which is consistent with the findings of the single-factor experiments. Within a voltage of 285–310 V and a temperature of 62–72 °C, the roughness was reduced below 0.05 μm. The model predicted a minimum roughness of 0.044 μm at 303.8 V and 66.2 °C, representing a 91.2% reduction compared to the initial surface.

For the material removal rate, the fitted quadratic regression model in the coded factors was

(4) $$MMR=53.90-2.01A-12.26B+0.0325AB+1.00A^{2}-1.24B^{2}$$

where *MMR* is the material removal rate.

The ANOVA results of the quadratic response surface model for the *MMR* are summarized in Table 3.

The model with *F* = 1239.52 and *p* < 0.0001 was also highly significant with a non-significant lack of fit with *p* = 0.3838, demonstrating excellent model adequacy. The linear terms of the voltage, with *F* = 158.73 and *p* < 0.0001, and temperature, with *F* = 5938.67 and *p* < 0.0001, were highly significant, whereas the *AB* term, with *F* = 0.0209 and *p* = 0.8893, was not significant.

The very small positive coefficient of AB and its large *p*-value indicates that the coupling between the voltage and temperature was negligible for the MMR. The partial derivatives were

(5) $$\frac{\partial MMR}{\partial A}=-2.01+2.00A+0.0325B$$

(6) $$\frac{\partial MMR}{\partial B}=-12.26-2.48B+0.0325A$$

At the optimal point, the partial derivatives were $\frac{\partial MMR}{\partial A}=-2.35$ and $\frac{\partial MMR}{\partial B}=-11.29$, confirming that temperature exerted a much stronger negative influence than voltage.

The regression performance of the *MRR* model was similarly evaluated. The fit statistics, including R^2^ = 0.9989, adjusted R^2^ = 0.9981 and predicted R^2^ = 0.9951, demonstrate excellent consistency between the experimental data and model predictions. The adequate precision value of 113.43 reflects a very strong signal relative to noise, confirming that the model response is well resolved within the investigated parameter range.

**Figure 6:**
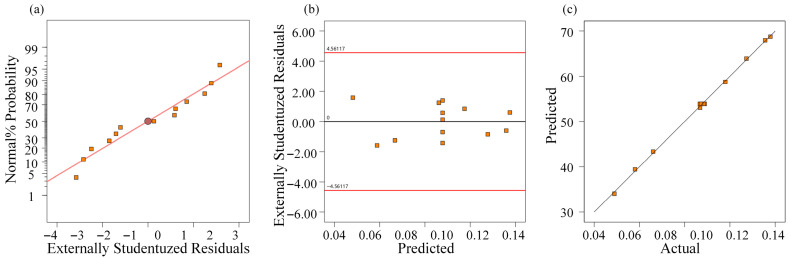
Residual diagnostics for the regression model of *MMR*: (**a**) normal probability plot of residuals, (**b**) residuals versus predicted, and (**c**) predicted versus actual.

The residual analysis shown in Figure 6 further supports the model’s reliability. No systematic trends or structural deviations were observed in the residual distribution, and the predicted values show close correspondence with the experimental results across the design space. These findings confirm the stability and predictive capability of the developed regression model for the material removal rate.

The 3D response surface and contour plots for the material removal rate are shown in Figure 5c,d. Under the conditions for the predicted optimal roughness, the predicted material removal rate was 58.90 mg·min^−1^, indicating that the minimum roughness could be achieved with a high material removal rate.

Three verification experiments were conducted at a voltage of 303.8 V, electrolyte temperature of 66.2 °C, polishing time of 5 min, and submersion depth of 7.5 cm. The results and error analysis are given in Table 4. The deviations between the experimental results and the model predictions were within 9.10% for the surface roughness and 1.09% for the material removal rate, confirming that the RSM model accurately predicted the surface roughness and material removal rate of GH3536 superalloy after PEP.

### 3.2. Surface Morphology of GH3536 Superalloy Before and After PEP

#### 3.2.1. Macroscopic and Microscopic Morphology

**Figure 7:**
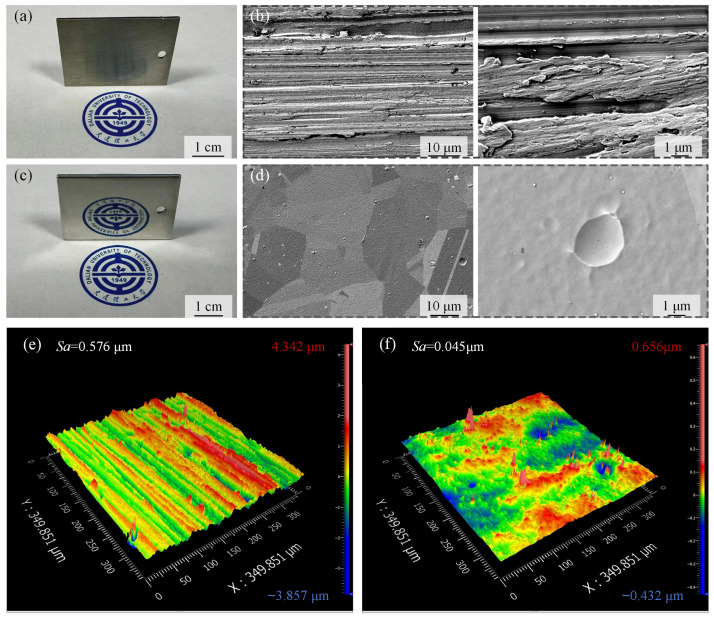
The morphology and 3D surface profiles of the GH3536 superalloy surface before and after PEP: (**a**) macroscopic image before PEP; (**b**) microscopic image before PEP; (**c**) macroscopic image after PEP; (**d**) microscopic image after PEP; (**e**) 3D surface profile before PEP; (**f**) 3D surface profile after PEP.

Based on the obtained optimal parameters, the surface morphologies of the samples before and after PEP were compared. As shown in Figure 7a, the optical image of the unpolished sample exhibited numerous surface defects. After PEP, these surface defects were eliminated, achieving a smooth, mirror-like finish (Figure 7c). Meanwhile, the microscopic morphologies were also compared, and the SEM images of the samples before and after PEP are presented in Figure 7b,d. The unpolished surface was rough and full of grooves and defects left by the abrasive paper. After PEP, the surface defects were removed, and the surface appeared uniformly smooth with a clear grain boundary. Additionally, as shown in the high-magnification image in Figure 7d, erosion pits and ripple-like features caused by the high-energy plasma bombardment inherent to the PEP mechanism were observed on the polished surface.

#### 3.2.2. 3D Surface Profile

The 3D surface profiles before and after PEP are shown in Figure 7e,f. The surface peak and valley values were reduced from 4.324 μm and −3.857 μm to 0.656 μm and −0.432 μm, respectively. The average surface roughness decreased from 0.576 μm to 0.045 μm. The peak heights and valley depths were markedly reduced, the surface undulation was diminished, and the surface became more uniform with significantly decreased roughness.

### 3.3. Chemical Composition

**Figure 8:**
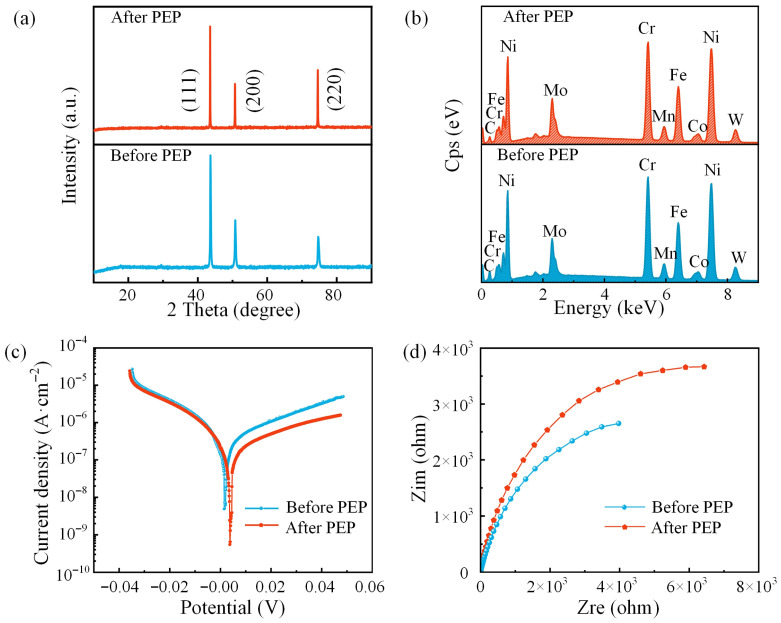
The chemical composition and electrochemical tests of samples before and after PEP: (**a**) XRD patterns of the sample surfaces before and after PEP; (**b**) EDS of the sample surfaces before and after PEP; (**c**) polarization curves of the samples before and after PEP; (**d**) Nyquist plots of the samples before and after PEP.

To investigate the phase transformation before and after PEP, XRD analysis was performed on the samples. The corresponding patterns are shown in Figure 8a. A comparison with the standard PDF cards confirmed the presence of diffraction peaks at 43.64°, 50.76°, and 74.68°, which correspond to the (111), (200), and (220) crystallographic planes, respectively. No new diffraction peaks were observed after PEP, which confirms that the polishing process did not alter the original FCC crystal structure of the GH3536 superalloy. However, the intensity of each diffraction peak was enhanced after PEP, indicating an increase in the surface crystallinity and a reduction in lattice defects.

Further investigation was conducted on the elemental composition of the surface before and after PEP. The EDS results are shown in Figure 8b and Table 5. It can be seen that the relative compositions of the elements C, Co, and Mn decreased, whereas Ni, Cr, Fe Mo, W, and Si increased after PEP. The different percentages of the elements were due to the selective dissolution by the electrolyte during the PEP process.

### 3.4. Corrosion Resistance

Corrosion resistance is a critical property of GH3536 superalloy, as it directly determines its reliability in aggressive environments. Therefore, electrochemical tests were conducted to evaluate and compare the corrosion behavior of the samples before and after PEP. Figure 8c shows the polarization curves of the samples measured in 3.5% aqueous NaCl solution. Table 6 lists the fitted parameters from the polarization curves. The corrosion potentials (*E*_corr_) of the samples before and after PEP were 0.002 V and 0.004 V, showing a slight positive shift. More significantly, the corrosion current density (*I*_corr_) decreased from 1.449 × 10^−7^ A·cm^−2^ to 8.192 × 10^−8^ A·cm^−2^, corresponding to a reduction of 43.46%, suggesting slower corrosion kinetics. Meanwhile, the polarization resistance (*R*_p_) increased from 5366 Ω·cm^2^ to 8249 Ω·cm^2^, representing an improvement of 53.72%. These results demonstrate a substantial enhancement in corrosion resistance after PEP. The Nyquist plots shown in Figure 8d further confirm this trend, as the PEP-treated sample exhibited a larger Nyquist semicircle, indicating higher charge transfer resistance and improved electrochemical stability.

The enhanced corrosion performance can be primarily attributed to the significant reduction in surface roughness after PEP, which decreased defect density and minimized localized corrosion initiation sites. A smoother surface facilitates the formation of a more uniform and stable passive film, improving resistance to chloride-induced breakdown. In addition, the EDS results show a noticeable increase in Ni and Mo contents after PEP. As Mo and Ni are corrosion-resistant elements in GH3536, their relative surface enrichment may enhance passive film stability and contribute to improved corrosion resistance.

In addition, the contact angle measurements in Table 7 show that the average contact angle increased from 60.0° to 70.9° after PEP treatment, indicating a moderate decrease in surface wettability. This change is consistent with the significant reduction in surface roughness and suggests a corresponding modification of the surface free energy state [32,33]. A smoother surface with reduced defect density and fewer high-energy sites may lower surface reactivity and decrease the tendency for localized chloride adsorption, thereby contributing to the suppression of localized corrosion initiation.

### 3.5. Subsurface Integrity Evaluation

To assess the influence of plasma electrolytic polishing on subsurface integrity, microhardness testing and residual stress measurement were performed.

**Figure 9:**
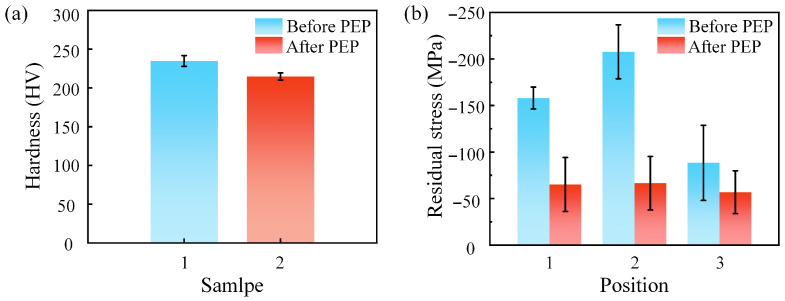
The microhardness residual stress tests of samples before and after PEP: (**a**) microhardness of the sample surfaces before and after PEP; (**b**) residual stress of the sample surfaces before and after PEP.

Figure 9a presents the Vickers hardness of the GH3536 surface before and after PEP. For each sample, 10 points were selected across the surface and measured using a Vickers hardness tester under a load of 0.5 kg. The surface hardness decreased from 234.8 HV after grinding to 214.8 HV following PEP, representing a slight reduction. This decrease was attributed to the removal of the work-hardened layer induced by prior metallographic grinding, which exposed the unaffected bulk material beneath the surface. The hardness after PEP treatment remained within the standard range, indicating that the polishing process did not adversely affect the material’s practical performance.

The residual stress measurements shown in Figure 9b indicate that the residual stress at three different surface locations decreased from −158 MPa, −207.7 MPa, and −88.5 MPa before PEP to −65.1 MPa, −66.6 MPa, and −56.9 MPa after PEP. Prior to PEP, the surface residual stress exhibited noticeable spatial variation. Although the magnitude of compressive residual stress decreased, the distribution became more uniform across the surface after PEP. During polishing, the surface experienced transient heating caused by ohmic heating and plasma discharge, followed by rapid cooling as the vapor–gas envelope collapsed and the surface recontacted the electrolyte. The repeated heating–cooling cycles facilitated partial relaxation and redistribution of the pre-existing deformation-induced residual stress. A more homogeneous compressive stress state helps reduce local stress gradients and potential stress concentration within the near-surface region, contributing to improved mechanical stability of the polished surface.

Overall, the PEP maintained subsurface integrity and improved near-surface mechanical stability.

## 4. Conclusions

In summary, we effectively improved the surface performance of GH3536 superalloy using plasma electrolytic polishing (PEP). We investigated the effects of the PEP process parameters on the surface roughness and material removal rate of GH3536 superalloy and optimized the parameters using response surface methodology. The surface morphology, phase composition, elemental composition and corrosion resistance of samples before and after PEP were characterized and analyzed. The main conclusions are as follows:

(1) The optimal PEP parameters for GH3536 were voltage of 303.8 V, electrolyte temperature of 66.2 °C, polishing time of 5 min, and submersion depth of 7.5 cm. Under these conditions, the surface roughness was reduced to 0.044 μm, representing a 91.2% reduction compared to the initial surface, and the material removal rate was 59.12 mg·min^−1^. Surface scratches and defects on the sample were removed, achieving a mirror-like finish.
(2) EDS analysis showed that the elemental composition remained essentially unchanged after PEP, with only slight variations in elemental proportions. XRD analysis indicated no new diffraction peaks after PEP, but the intensity of each diffraction peak increased.
(3) The corrosion resistance was enhanced, with *R*_p_ rising by 53.72% and *I*_corr_ decreasing by 43.46%. The combined reduction in surface roughness and enhancement in corrosion resistance can extend the durability of components operating at high temperatures and in aggressive environments.
(4) PEP slightly reduced the surface hardness due to removal of the work-hardened layer but remained within standard limits. The compressive residual stress decreased moderately but became more uniform, reducing local stress gradients. These changes demonstrate that PEP maintained subsurface integrity and improved near-surface mechanical stability.

This study evaluated corrosion behavior through short-term electrochemical testing and confirmed improved electrochemical performance. Future work will extend the assessment to prolonged and cyclic exposure conditions to further clarify the long-term durability of the PEP-treated surface.

## Figures and Tables

**Table 1:** The experimental results of the central composite design.

Test Serial Number	Voltage (V)	Electrolyte Temperature (°C)	Surface Roughness ***Ra*** (μm)	Material Removal Rate (mg·min^−1^)
1	312.5	70	0.047	54.43
2	350.0	60	0.092	63.65
3	259.5	70	0.075	58.98
4	275.0	80	0.070	43.04
5	312.5	84	0.087	34.47
6	312.5	70	0.049	53.96
7	312.5	70	0.049	53.37
8	312.5	56	0.056	68.94
9	312.5	70	0.048	54.15
10	275.0	60	0.068	67.79
11	365.5	70	0.131	53.40
12	350.0	80	0.126	39.03
13	312.5	70	0.048	53.61

**Table 2:** The ANOVA results of the quadratic response surface model for *Ra*.

Source	Sum of Squares	df	Mean Square	*F*-Value	*p*-Value
Model	0.0101	5	0.0020	811.92	<0.0001
*A*-Voltage	0.0032	1	0.0032	1278.76	<0.0001
*B*-Temperature	0.0008	1	0.0008	317.1	<0.0001
*AB*	0.0003	1	0.0003	103.34	<0.0001
*A* ^2^	0.0053	1	0.0053	2156.54	<0.0001
*B* ^2^	0.0010	1	0.001	408.58	<0.0001
Residual	1.73 × 10^−5^	7	2.48 × 10^−6^		
Lack of Fit	1.21 × 10^−5^	3	4.05 × 10^−6^	3.11	0.1507
Pure Error	5.20 × 10^−6^	4	1.30 × 10^−6^		
Cor Total	0.0101	12			

**Table 3:** The ANOVA results of the quadratic response surface model for the *MMR*.

Source	Sum of Squares	df	Mean Square	*F*-Value	*p*-Value
Model	1255.86	5	251.17	1239.52	<0.0001
*A*-Voltage	32.17	1	32.17	158.73	<0.0001
*B*-Temperature	1203.39	1	1203.39	5938.67	<0.0001
*AB*	0.0042	1	0.0042	0.0209	0.8893
*A* ^2^	6.96	1	6.96	34.36	0.0006
*B* ^2^	10.73	1	10.73	52.96	0.0002
Residual	1.42	7	0.2026		
Lack of Fit	0.7065	3	0.2355	1.32	0.3838
Pure Error	0.7119	4	0.178		
Cor Total	1257.27	12			

**Table 4:** The verification experiment results.

Test Serial Number	Surface Roughness *Ra* (μm)	Relative Error	Material Removal Rate (mg·min^−1^)	Relative Error
1	0.045	2.27%	58.63	0.45%
2	0.044	0	59.36	0.78%
3	0.047	9.10%	58.26	1.09%

**Table 5:** The elemental composition of the sample surfaces before and after PEP.

	C	Si	Cr	Mn	Fe	Co	Ni	Mo	W
Before PEP (%)	6.36	0.23	20.87	0.65	16.78	1.76	44.76	7.70	0.90
After PEP (%)	4.54	0.26	21.15	0.64	16.94	1.70	45.26	8.53	0.99

**Table 6:** The fitting results of the polarization curves.

	*E*_corr_ (V)	*I*_corr_ (A·cm^−2^)	Anode Tafel Slope *β*_a_	Cathode Tafel Slope *β*_c_	*R*_p_ (Ω·cm^2^)
Before PEP	0.002	1.449 × 10^−7^	0.158	0.059	5366
After PEP	0.004	8.192 × 10^−8^	0.094	0.066	8249

**Table 7:** The contact angle of GH3536 superalloy surface before and after PEP.

Contact Angle (°)	Position 1	Position 2	Position 3	Average
Before PEP	58.2	61.5	60.4	60.0
After PEP	68.4	71.6	72.7	70.9

## Data Availability

The original contributions presented in this study are included in the article. Further inquiries can be directed to the corresponding authors.
